# Doubled Haploid Production in *Cucurbita pepo* L. Through Ovary Culture

**DOI:** 10.3390/plants14243733

**Published:** 2025-12-08

**Authors:** Ana García-Pérez, Malen Escánez, Sandra Gil, Alejandro Miralles-Rodríguez, Santiago Vilanova, Francisco Bermúdez, Edgar García-Fortea

**Affiliations:** 1 Seeds for Innovation S.L., Edificio PITA, Lab 43, Carretera Sacramento s/n, 04120 Almería, Spain; malen.escanez@beyond-seeds.com (M.E.); sandra.gil@beyond-seeds.com (S.G.); alejandro.miralles@beyond-seeds.com (A.M.-R.); francisco.bermudez@beyond-seeds.com (F.B.); edgar.garcia@beyond-seeds.com (E.G.-F.); 2 Instituto Universitario de Conservación y Mejora de la Agrodiversidad Valenciana, Universitat Politècnica de València, Camí de Vera s/n, 46022 Valencia, Spain; sanvina@upvnet.upv.es

**Keywords:** embryogenesis, haploid induction, swollen ovules, spontaneous diploidization, gametic regeneration, *Cucurbitaceae*, zeatin riboside

## Abstract

Gynogenesis offers a promising route for doubled haploid (DH) production in *Cucurbita*, yet efficient protocols remain scarce. This study established a reproducible ovary culture system for *Cucurbita pepo* and evaluated zeatin riboside (ZR) as an alternative cytokinin. Ovaries collected at anthesis and one day before were cultured to screen nine media with different cytokinin–auxin combinations. Subsequently, four optimized ZR-based formulations were evaluated. Both floral stages showed morphogenic activity, but embryo formation occurred almost exclusively in pre-anthesis ovaries. Among ZR treatments, E6.1 (1 mg·L^−1^ ZR + 3 mg·L^−1^ NAA, 30 g·L^−1^ sucrose) achieved the highest embryogenic output (approximately 97 embryos per 100 explants), while high-sucrose media (120 g·L^−1^) induced abundant swollen ovules but poor conversion, suggesting that excessive osmotic pressure promotes morphogenesis but hampers embryogenic transition. In total, 415 embryos were obtained, and 52 regenerants were analyzed by flow cytometry, confirming haploid, diploid, and mixoploid plants and evidencing spontaneous chromosome doubling during in vitro development. A categorical A–D scoring system enabled early prediction of embryogenic potential. This represents the first successful application of ZR in cucurbit gynogenesis and highlights its value as a biologically compatible cytokinin for DH production. The findings open new avenues for testing ZR-based formulations in other *Cucurbita* species under different auxin and sucrose regimes.

## 1. Introduction

Zucchini (*Cucurbita pepo* L.), along with other squashes such as *Cucurbita maxima* and *Cucurbita moschata*, belongs to the *Cucurbitaceae* family and represents a group of horticultural crops of major economic importance worldwide. In 2023, global cucurbit production exceeded 23 million tons [1]. In Spain, zucchini ranks as the third most widely cultivated greenhouse crop by area, with over 11,000 hectares and an annual production surpassing 590,000 tons [2]. This productive relevance underscores the need to optimize biotechnological tools that can accelerate breeding and varietal improvement programs.

The development of doubled haploid (DH) lines is a key strategy for rapidly achieving homozygosity within a single generation, thereby significantly reducing the time required to develop stable varieties [3,4,5]. In cucurbits, several approaches have been implemented for haploid production, including androgenesis, gynogenesis, and parthenogenesis [6,7]. In the *Cucumis* genus (cucumber and melon), the culture of anthers, ovules, and unpollinated ovaries has yielded particularly successful results [8,9,10]. For instance, in cucumber, fully homozygous lines have been obtained through haploid embryos induced from immature ovaries [10].

In *C. pepo*, by contrast, the application of such methodologies remains very limited. Strategies such as the use of irradiated pollen [11], anther culture [12,13], and gynogenesis through immature ovule culture [14,15,16] have been explored, but results have been highly variable and genotype-dependent. In other cucurbit crops, such as cucumber and melon, pollination with γ-irradiated pollen followed by in vitro rescue of parthenogenetic embryos has also been successfully used to generate doubled haploid lines [17,18,19]. However, these protocols rely on Co^60^ irradiation facilities, specific radiation-safety procedures, and dedicated embryo-rescue pipelines, which limits their routine implementation in many breeding programs. Within this context, alternative gynogenetic approaches that do not require pollen irradiation are particularly attractive. Gémesné-Juhász et al. [20] demonstrated the formation of embryos from immature zucchini ovaries cultured in vitro, albeit with low efficiency. A more recent review by Domblides et al. [21] supports the potential of ovary culture in *C. pepo*, while acknowledging the lack of efficient and reproducible protocols. In contrast, in *C. maxima* and *C. moschata*, ovary culture has shown greater consistency and efficacy in embryogenic induction [22,23,24]. This technical gap in *C. pepo* highlights the need to optimize and adapt species-specific protocols.

Among the methods tested in cucurbits, the culture of slices of unpollinated ovaries offers several advantages over the manipulation of individual ovules. This approach minimizes damage to the female gametophyte, allows for uniform application of stress treatments, and facilitates the simultaneous induction of multiple female gametes [6,22]. Several critical factors influence the efficiency of this process. For instance, ovaries harvested before anthesis, when female gametophytes are still at juvenile stages, exhibit higher embryogenic potential [25,26]. However, favorable responses have also been reported in flowers collected at anthesis, as observed in melon (*Cucumis melo*) [8,27] or zucchini (*C. pepo*) [14,15,16]. These findings suggest that the optimal embryogenic response window may vary depending on the species, genotype, and in vitro protocol conditions. Another key factor is the application of stress pre-treatments, such as incubation at low temperatures (4 °C for 24–72 h) or heat (33–35 °C in darkness), which can trigger cellular reprogramming toward embryogenic pathways [28,29]. Additional factors, such as culture medium composition (sucrose levels, salts, and growth regulators), also influence the embryogenic response [30,31].

In cucurbits, classical media formulations typically use auxins such as 2,4-D or NAA combined with cytokinins like BAP or kinetin [6,7]. These combinations often result in genotype-dependent and suboptimal responses. Therefore, alternative cytokinins, such as ZR, have emerged as promising candidates. In eggplant (*Solanum melongena*), García-Fortea et al. [32] established a ZR-based (2 mg·L^−1^) anther culture protocol that enabled the regeneration of DH plants. Similarly, Dehkehan et al. [33] reported improved DH seedling production using ZR (1 mg·L^−1^) with NAA (3 mg·L^−1^). Earlier, Bennici and Cionini [34] also noted that both ZR and zeatin (Z) stimulated early embryogenic development in vitroin vitro in *Phaseolus coccineus*. Although ZR has been identified as an endogenous cytokinin in cucurbits [35], its application in DH induction protocols for *Cucurbita* has not yet been evaluated. Its endogenous occurrence in this family suggests that ZR may promote a more compatible morphogenic response than synthetic cytokinins such as BAP or TDZ.

Considering these gaps and the promising role of ZR in other species, this study aimed to establish an efficient protocol for generating DH zucchini lines (*C. pepo*) through unpollinated ovary culture. For the first time in this species, we evaluated the effects of a ZR-based hormonal combination, previously effective in *S. melongena*, on embryogenic induction. We assessed the influence of floral stage, tested various culture media reported in cucurbits and other crops, and optimized cytokinin formulations to improve embryo induction and regeneration. The double haploid status of the regenerated lines was confirmed by applying flow cytometry. This study expands the current toolkit for in vitro haploid induction in cucurbits and contributes to the development of reliable DH production systems in *C. pepo*.

## 2. Results

### 2.1. Developmental Stages of Ovary-Derived Explants

**Figure 1:**
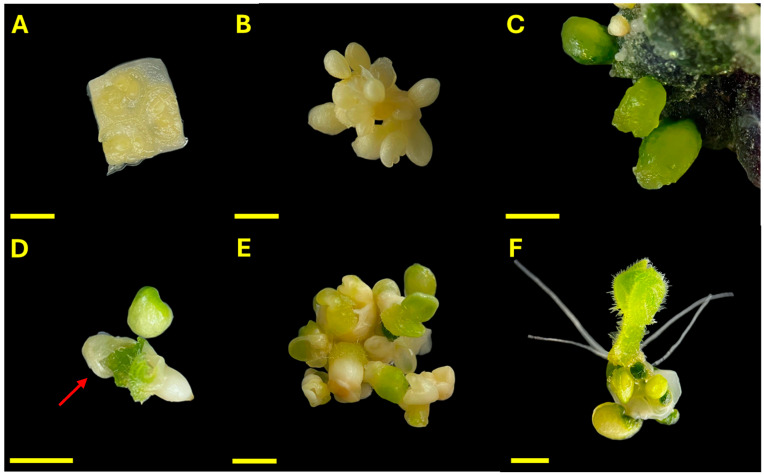
Progressive development of *Cucurbita pepo* ovary-derived explants under in vitro conditions. (**A**) Ovary explant at day 0; (**B**) Ovule swelling after 7 days of dark incubation at 35 °C; (**C**) Emergence of globular ELSs; (**D**) Heart-shaped and cotyledonary embryo emerging from within the ovule; the red arrow indicates residual ovular integuments partially enclosing the structure; (**E**) Multiple embryo stages present on a single explant; (**F**) Fully differentiated cotyledonary embryo with visible roots. Scale bars = 0.25 mm.

The progression of embryo development and plant regeneration from unpollinated ovary cultures of *C. pepo* is illustrated in Figure 1. Ovarian explants were cultured under dark conditions at 35 °C for 7 days (Figure 1A). During this period, the ovules exhibited visible swelling and yellowing of integuments, indicating an early response to inductive treatment (Figure 1B). Upon transfer to light conditions, the ovules gradually turned green, and some explants develop dark green callus regions. Embryo-like structures (ELSs) with a globular morphology emerged within 2–3 weeks of culture (Figure 1C). By day 45, well-defined ELSs developed from the ovary tissues, showing characteristic heart-shaped and cotyledonary forms (Figure 1D). Occasionally, embryos were observed emerging fully formed from within the ovular structure, with residual integument-like tissues partially enclosing them, resembling the morphology of a developing seed. Frequently, multiple embryos at different stages of development, globular, heart, torpedo, and cotyledonary, were observed on the same explant (Figure 1E). Fully developed cotyledonary embryos with visible roots were obtained (Figure 1F).

**Figure 2:**
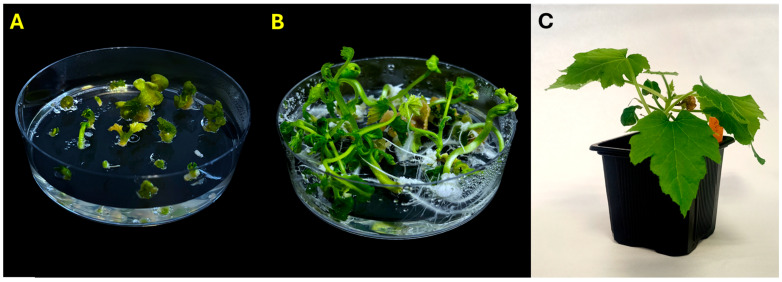
Regeneration and acclimatization of *C. pepo* embryos. (**A**) Embryos transferred to regeneration medium; (**B**) Fully developed plantlets; (**C**) Acclimatized plant growing in soil under controlled conditions.

Mature embryos were transferred to regeneration medium (Figure 2A), where they successfully developed shoots and roots (Figure 2B), and were later acclimatized to soil under controlled conditions (Figure 2C).

### 2.2. Initial Screening of Culture Media

#### 2.2.1. General Overview of Species and Floral Stage Response

An initial screening was performed in two species of *Cucurbita*: *C. pepo* (zucchini) and *C. maxima* (pumpkin), using ovaries collected either one day before anthesis (1 DBA) or at anthesis. No response was observed in *C. maxima* under any tested condition. Although some explants exhibited swelling and cork-like tissue development (Supplementary Figure S1), these responses did not progress to callus or embryo formation.

In contrast, *C. pepo* exhibited embryogenic responses at both floral stages, which were classified into four types (A–D; see Materials and Methods). The developmental stage significantly affected induction efficiency: explants collected at 1 DBA showed a markedly higher frequency of type D responses, whereas those collected at anthesis produced virtually no embryos (Table 1). A few exceptional cases were observed in anthesis-derived explants cultured on medium E6.2, which yielded three embryos out of one hundred explants; however, these isolated events were not considered biologically relevant. A chi-squared test confirmed a significant association between floral stage and response type (χ^2^ = 22.6, df = 3, *p* < 0.001), with a Cramer’s V of 0.12, indicating a small-to-moderate effect size. Based on these results, subsequent experiments focused exclusively on *C. pepo* explants collected at 1 DBA, which consistently exhibited embryogenic competence.

#### 2.2.2. Comparison of Morphological Responses Across Culture Medium

The morphogenic response of *C. pepo* ovary explants collected 1 DBA was evaluated across nine culture media. Based on the previously defined response categories (A–D), a chi-squared test confirmed a significant association between culture medium and morphological response type (χ^2^ = 256.31, df = 21, *p* < 0.001), with a Cramer’s V of 0.33, indicating a moderate effect size.

**Figure 3:**
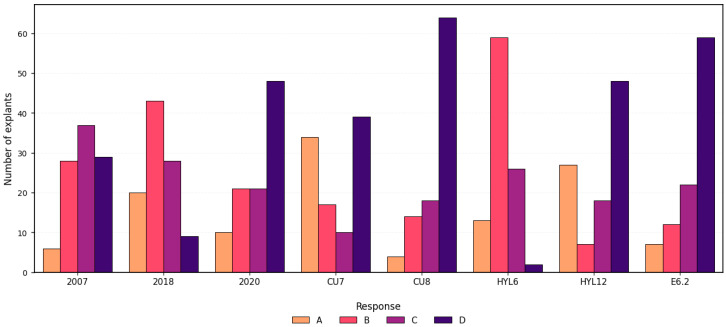
Distribution of morphological response types in *Cucurbita pepo* ovary explants across eight culture media. Responses were categorized as A: no response; B: callus; C: callus with swollen ovules; D: swollen ovules. Bars represent the number of explants per category (n = 100 per medium). Data correspond to explants collected one day before anthesis (1 DBA).

Among the tested media, E6.2 and CU8 showed the highest frequencies of type D responses (59% and 64%, respectively) indicating a strong embryogenic potential. In contrast, HYL6 and 2018 were dominated by type B responses, with minimal occurrence of type D. HYL12 and CU7 exhibited similar profiles, characterized by high frequencies of types D and A, along with low callus formation tendency. Medium 2007 and medium 2020 showed no dominant response type, as categories B, C, and D displayed similar frequencies; however, 2020 demonstrated a higher propensity for type D responses (Figure 3). Post hoc pairwise comparisons with Bonferroni correction, along with hierarchical clustering, confirmed the statistical differences between media and identified groups with similar response patterns (Supplementary Figures S2 and S3) further supported these trends. Although both HYL6 and HYL12 contained TDZ, their responses differed markedly.

#### 2.2.3. Embryo Production Across Medium

Embryo production displayed a highly skewed distribution, with most explants producing no structures and only a few generating multiple embryos. Post hoc comparisons using Dunn’s test with Bonferroni correction revealed that E6.2 produced significantly more embryos than all other media (50 embryos from 29 explants). The medium 2007 showed intermediate performance (27 embryos), whereas the remaining media exhibited similarly low yields, with no significant differences among them.

In terms of biological efficiency, E6.2 induced embryos in 29% of explants. Medium 2007 achieved 17% efficiency, while all other media induced embryos in fewer than 5% of explants. These results confirm E6.2 as the most effective medium under the tested conditions, combining both a high induction frequency and a moderate embryo yield per explant.

#### 2.2.4. Association Between Morphological Response and Embryo Yield

To assess whether the initial morphological response predicted embryogenic competence, embryo production was compared across response types. A Kruskal–Wallis test revealed a highly significant effect of response type on both total embryo counts (H = 37.68, *p* < 0.001) and the number of embryogenic explants (H = 37.43, *p* < 0.001). Post hoc comparisons using Dunn’s test with Bonferroni correction showed that responses A and B did not differ significantly (*p* = 1.0), but both were significantly lower than responses C and D (*p* < 0.021). No statistical difference was detected between C and D (*p* = 0.49) in terms of final embryo production.

**Figure 4:**
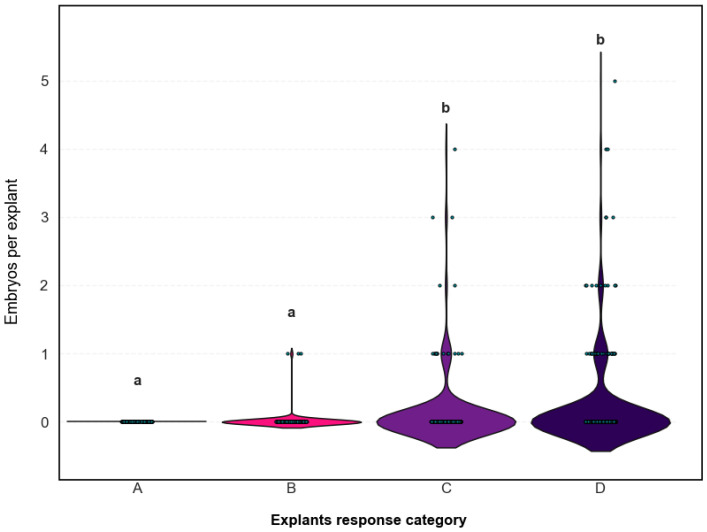
Distribution of total embryo production per explant in *C. pepo* according to initial morphological response type. Each violin plot represents the number of embryos formed per explant (*y*-axis) for each response category (A: no response; B: callus; C: callus with swollen ovules; D: swollen ovules). Gray dots represent individual explants (n = 800 total; 100 per medium). The width of each violin reflects the frequency of explants at a given embryo count. Lowercase letters (a, b) indicate statistically significant differences between response categories (*p* < 0.05).

These results confirm the predictive value of the morphological classification: explants showing ovule induction (C and D) were significantly more likely to produce embryos, whereas those exhibiting no response or callus alone (A and B) displayed minimal embryogenic activity (Figure 4).

### 2.3. Comparative Analysis of Zeatin Riboside-Based Media for Ovule Induction and Embryogenic Response

#### 2.3.1. Induction of Swollen Ovules (SOs) in Zeatin Riboside-Based Media

Given that medium E6.2, which contains ZR, showed the highest embryogenic response during the initial screening, we further investigated the role of this cytokinin by testing four ZR-based formulations. The ability of four culture media to induce swollen ovules (SOs) in unpollinated ovaries of *C. pepo* was evaluated.

All treatments promoted high response rates, with the percentage of responsive explants ranging from 62.3% in E6 to 84.9% in E6.2 (Table 2). The number of induced structures per explant differed significantly among media (Kruskal–Wallis, H = 28.58, *p* < 0.0001), with mean values ranging from 4.00 ± 4.18 in E6, to 5.64 ± 3.68 in E6.1, 6.01 ± 4.16 in E6.3 and 6.09 ± 3.54 in E6.2. When normalized per 100 explants, E6.2 produced the highest number of swollen ovules (609), followed by E6.3 (601), E6.1 (564), and E6 (400). Despite the broadly high induction rates, the relatively large standard deviations reveal a high degree of variability among explants, a common feature in ovary cultures. Overall, E6.2 demonstrated the highest induction capacity.

#### 2.3.2. Embryo Production in ZR-Based Media

**Figure 5:**
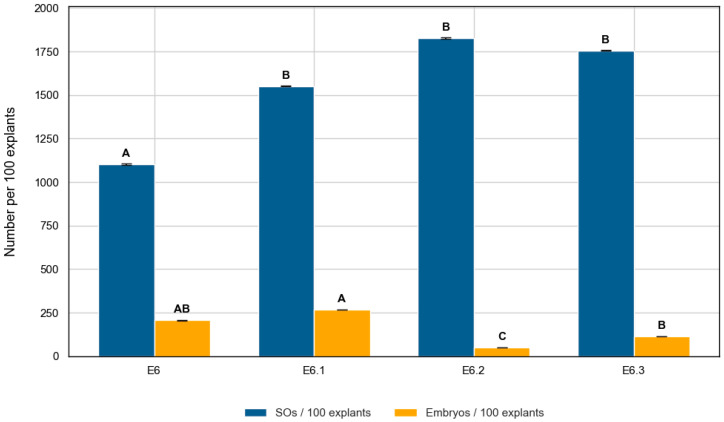
Swollen ovule (blue) and embryo (orange) induction per 100 *C. pepo* explants cultured in four ZR-based media. Bars represent mean ± SD. Different letters denote significant differences among treatments (Dunn’s test, *p* < 0.05).

Embryogenic output was assessed using three parameters: number of embryos per explant, percentage of embryogenic explants, and conversion rate (embryos per SO). The number of embryos per explant differed significantly among media (Kruskal–Wallis, H = 43.78, *p* < 0.0001). Pairwise comparisons revealed that E6.1 produced significantly more embryos than E6.2 (*p* < 0.0001) and E6.3 (*p* = 0.0006), while E6 also outperformed E6.2 (*p* = 0.0006) (Table 3). In general, embryogenic efficiency followed the trend E6.1 ≈ E6 > E6.3 > E6.2. Despite inducing the highest number of SOs, E6.2 yielded the lowest embryo production within the four ZR-based media (Figure 5).

Conversion efficiency also varied significantly among treatments (H = 42.88, *p* < 0.0001), with values of 18.7% (E6), 17.2% (E6.1), 6.4% (E6.3), and 2.7% (E6.2). As illustrated in Figure 5, SO induction and embryo production did not follow the same trend across media. The relatively high standard deviations indicate substantial variability among explants. E6.1 combined high responsiveness, favorable conversion efficiency, and a greater proportion of embryogenic explants compared to the other media.

Although at a global level SO induction did not predict embryo yield, specific morphogenic response types were associated with embryo formation. To further clarify the source of variation, the relationship between morphogenic response type and embryo production was examined (Table A1). Only explants classified as C or D produced embryos, with significantly higher counts than types A or B (H = 69.56, *p* < 0.001). High-producing explants (≥4 embryos) were almost exclusively associated with type D responses (χ^2^ = 17.45, *p* = 0.011). No significant differences were observed between types C and D within the subset of productive explants (U = 2.0, *p* = 0.455). In general, embryo formation was restricted to explants showing advanced morphogenic responses, with type D being the most frequent among those classified as embryogenic.

### 2.4. Determination of Ploidy Level and Acclimatization

**Figure 6:**
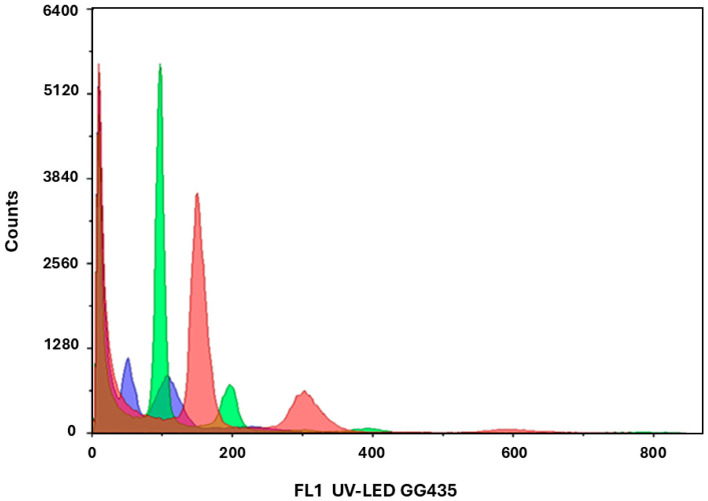
Representative flow cytometry histograms of *C. pepo* regenerants showing haploid (blue), diploid (green), and triploid (red) DNA peaks. The diploid peak was normalized to 100 on the relative fluorescence axis.

Across all experimental treatments, approximately 415 regenerants were obtained. A subset of 52 plantlets, representing those that reached a stable vegetative stage, was analyzed by flow cytometry. Among them, five plants displayed haploid (n) DNA profiles, eight were chimeric or had ploidies higher than diploid (>2n), and the remainder were diploid (2n). Representative histograms of haploid, diploid, and triploid plants are shown (Figure 6). Several plants initially identified as haploid later exhibited diploid profiles upon reanalysis, consistent with spontaneous genome duplication during in vitro development. During the isolation and conversion stages, some regenerants were lost due to physiological or microbial stress. Nevertheless, several plants were successfully acclimatized, and eight confirmed regenerants are currently established under greenhouse conditions. Morphologically, all greenhouse plants displayed a very similar appearance (typical uniform green zucchini phenotype) and no distinguishing traits were observed among them. The main differences related to plant vigor (Supplementary Figure S4), as low-vigor plants failed to set fruit under greenhouse conditions. Only the vigorous plants set fruit upon self-pollination, so seeds were obtained for 6 of the 8 individuals, effectively immortalizing these lines.

## 3. Discussion

A clear contrast was observed between the two *Cucurbita* species: only *C. pepo* responded with embryo formation, whereas *C. maxima* showed no morphogenic activity under the same conditions, including media previously reported as effective for related genotypes [23,36]. Although effective ovary culture protocols have been described for *C. maxima* and *C. moschata* [23,24], genotypic and physiological differences likely explain the lack of response observed in our study [7,37]. Kurtar et al. [36] also noted that gynogenic efficiency varies widely among *Cucurbita* species and lines, emphasizing the need for genotype-specific optimization. In our experiments, *C. pepo* responded in all tested media, even in formulations originally developed for *C. maxima*, reaching up to 97 embryos per 100 explants in the best-performing medium (E6.1), which exceeds the 27 embryos per 100 explants reported by Shalaby for *C. pepo*-specific media [16].

In *C. pepo*, both floral stages, pre-anthesis (1 DBA) and anthesis, showed morphogenic activity; however, embryo formation occurred almost exclusively from ovaries collected at 1 DBA. Only three embryos were obtained from anthesis explants cultured on medium E6.2, indicating that under highly favorable osmotic and hormonal conditions, some residual embryogenic potential may persist even at later floral stages. This pattern is consistent with observations in other cucurbits, such as cucumber, melon, and pumpkin, where harvesting prior to anthesis enhances gynogenic induction [7,10,38,39]. Younger female gametophytes retain greater developmental plasticity and are more prone to reprogram toward embryogenesis [26], which could explain the superior response of the 1 DBA stage. Comparable trends have been described in other taxa, including Liliaceae (*Allium cepa*) [25] and Cactaceae (*Hylocereus undatus*) [30], where ovule immaturity correlates with higher morphogenic competence. Nevertheless, some *Cucurbita* species may also respond to anthesis. For instance, gynogenetic haploids have been obtained from ovaries collected at anthesis in cucumber and melon [20,27,40], indicating that the optimal induction window varies with genotype and species.

In *Cucurbita pepo*, efforts to obtain haploids have mainly focused on androgenesis and gynogenesis, both showing modest efficiency and strong genotype dependence [12,13,16]. Pollen-irradiation-induced parthenogenesis has also been explored, but dihaploidization frequencies remain low [11,41]. In zucchini, irradiated-pollen protocols have yielded haploid plants from parthenogenetic embryos, with embryo stage strongly affecting the outcome and a total of 93 haploid plantlets recovered [11]. In melon, parthenogenetic embryo rescue from γ-irradiated pollen has enabled the generation of 109 doubled-haploid lines from segregating populations [19]. In cucumber, reported averages of embryos that developed into plants ranged from 0.3 to 0.9 plants per 100 seeds depending on the genotype [17]. These values, although apparently low, refer only to rescued parthenogenetic embryos and are not directly comparable to total embryo induction because they depend on the morphological criteria used to assign embryo origin. Although effective, these systems require extensive seed and embryo screening and access to irradiation facilities, which restricts their wider use. In contrast, the simpler logistics and competitive efficiency of ovary culture make it a practical alternative for DH production in cucurbits. Most gynogenetic studies in *C. pepo* have relied on the culture of unfertilized ovules, achieving induction efficiencies rarely above 11–28% [15,16]. Although widely used, these approaches are technically demanding and often affected by tissue damage and losses during ovule excision. In contrast, ovary slice culture offers a simpler and faster alternative, allowing simultaneous exposure of multiple ovules to the culture medium while preserving their structural integrity. Despite its potential, this approach has been scarcely investigated in *C. pepo*, with few reports available, including that of Gémesné-Juhász et al., who obtained 10–15 embryos per ovary [20]. In other cucurbits, however, ovary culture has yielded comparable or higher efficiencies, producing 12 embryogenic structures and 2 plantlets per 100 ovary slices in *C. maxima* and 39 embryogenic structures and 10 plantlets in *C. moschata* [23], and up to 36% embryo induction in *C. moschata* [24]. Within this framework, the present study demonstrates that ovary culture in *C. pepo* is a technically simple, reproducible, and efficient system for gynogenetic induction, capable of producing consistent embryogenic responses and viable haploid and doubled-haploid regenerants.

To objectively describe these responses, a four-level categorical system (A–D) was implemented, distinguishing non-responsive explants (A), undifferentiated callus (B), callus with swollen ovules (C), and swollen ovules (D). Only categories C and D produced embryos, demonstrating that this visual scoring approach serves as a reliable early indicator of embryogenic potential. Morphological classification is widely used in plant tissue culture, although most studies apply binary systems, such as the distinction between embryogenic and non-embryogenic callus in *Cannabis sativa* [42] or responsive versus non-responsive cell lines in *Pinus koraiensis* [43]. Similarly, in maize (*Zea mays*) was described the type I/type II callus distinction, linking morphology to embryogenic competence [44]. Likewise, Martínez-López et al. [45] proposed a three-level ordinal scale for *Capsicum* organogenesis, illustrating the broader usefulness of visual categorical scoring. In this context, the A–D scale developed for *C. pepo* refines existing approaches by characterizing the morphogenic continuum from non-responsive to fully embryogenic explants and providing a practical tool for the preliminary evaluation of gynogenetic culture.

The analysis of morphogenic responses revealed a strong association between morphogenic response type and embryogenic outcome. Embryos were produced exclusively by explants classified as types C and D, characterized by the presence of SOs, whereas explants showing callus proliferation or no response (types A and B) exhibited no embryogenic activity. These findings, consistent with the statistical analyses reported above, indicate that morphogenic response type is a reliable early predictor of embryogenic potential. The association of type D explants with embryo formation further suggests that embryogenesis in *C. pepo* is likely derived from female gametophytic tissues rather than from somatic callus proliferation.

However, high ovule induction did not always translate into higher embryo production. Similar trends were reported by Zou et al. [23] and Min et al. [24], who observed that high rates of swollen ovules or greening structures did not necessarily translate into higher embryo production, reflecting variability in regenerative capacity among genotypes and developmental stages. Histological evidence summarized by Dong et al. [7] indicates that gynogenic embryos in cucurbits can arise from different tissues depending on the species. In *C. pepo*, embryos have been shown to develop directly from embryo-sac cells [14,46], and direct embryo formation from unpollinated ovules has also been documented in cucumber (*Cucumis sativus*) [40]. In our regenerants, several embryos emerged from within the ovary, with some retaining residual tegumentary tissues adhering to the young leaves. These structures, although not true seed coats due to the absence of fertilization, reinforce the interpretation that embryo development originated from internal ovule tissues rather than from somatic callus proliferation. Beyond cucurbits, cross-species evidence reviewed by Bohanec [26] indicates that excised ovules can continue embryo-sac maturation in vitro and that several gametophytic cells, such as the egg cell, synergids, antipodal cells, or non-fused polar nuclei, may theoretically initiate haploid embryos. However, most reports identify the egg cell as the predominant source of haploid embryos across taxa. Consistently, Kurtar and Seymen [37] observed that in *C. moschata* and *C. maxima*, firm and greenish calli serve only as transient support for embryoids, while differentiation occurs on distinct ovule-like protrusions at the explant surface. Altogether, these findings indicate that the type D response observed in *C. pepo* corresponds to a genuine gynogenetic, gametophytic pathway, consistent with previous descriptions in cucurbits.

The response to classical regulators followed previously described trends. Moderate auxin–cytokinin ratios promoted morphogenic stability, whereas excessive concentrations induced callogenesis or inhibited morphogenic response [7,39,47,48]. In our assays, media combining multiple regulators or containing high hormone levels, such as 2018 (4 mg·L^−1^ BAP + 0.05 mg·L^−1^ NAA + 0.1 mg·L^−1^ TDZ), mainly led to callus proliferation, while CU7 (5 mg·L^−1^ 2,4-D + 5 mg·L^−1^ kinetin) resulted in a high proportion of non-responsive explants. These results reinforce that excessive or complex hormonal interactions disrupt developmental balance [36]. In contrast, the 2007 formulation (1 mg·L^−1^ 2,4-D + 1 mg·L^−1^ kinetin) produced the only notable embryogenic response, reaching about 17 embryogenic explants per 100 cultured. These observations are consistent with those of Kurtar and Seymen [37], who reported that excessive regulator concentrations disrupt morphogenic stability.

In general, all ZR-based media performed very well, confirming the high responsiveness of this cytokinin in *C. pepo*. Formulations containing only ZR, such as E6 and E6.3, promoted strong morphogenic activity and embryo formation, although the E6.1 medium (1 mg·L^−1^ ZR + 3 mg·L^−1^ NAA, 30 g·L^−1^ sucrose) yielded the highest number of embryos per explant. This enhanced response suggests that a balanced cytokinin/auxin ratio may play a key role in promoting both ovule enlargement and subsequent embryogenesis. This represents the first documented application of ZR in gynogenesis protocols for *Cucurbita*, highlighting its potential as natural cytokinin capable of supporting more physiologically coherent responses than synthetic regulators such as BAP or TDZ. Comparable results have been reported in other species, where ZR enhanced regeneration and doubled haploid production in *S. melongena* and *P. coccineus* [32,33,34]. In light of these results, ZR appears to play a key role in the response of the ‘Victoria’ hybrid; however, this has so far been demonstrated in a single genotype, and it will be important to test ZR-based formulations in additional *C. pepo* backgrounds. In eggplant, ZR-based protocols show relatively low genotype dependence [32], which makes it particularly interesting to investigate whether a similar pattern can be observed in *C. pepo*.

The best embryogenic outcomes were obtained in the ZR-based media containing low to moderate sucrose levels (15–30 g·L^−1^), particularly in E6 and E6.1, whereas all media with elevated sucrose (E6.2, HYL12, and CU8, each containing 120 g·L^−1^) showed a pronounced morphogenic response but poor embryo conversion. This suggests that high osmotic potential promotes tissue swelling and the formation of swollen ovules yet impedes the transition toward organized embryogenesis. Comparable patterns have been described in *C. pepo* and *Theobroma cacao* [16,49] where moderate sucrose concentrations yielded the best induction and embryo development rates, while other species such as *H. undatus* [30] and *Allium* spp. [50] exhibit optimal induction at higher sucrose concentrations. This contrast indicates that osmotic pressure interacts synergistically with hormonal balance to modulate the morphogenic potential of explants.

Flow cytometric analysis confirmed the presence of haploid (n), diploid (2n), and mixoploid (n + 2n) plants demonstrating that embryo development in *C. pepo* originated from gametophytic cells. The occurrence of spontaneous chromosome duplications during in vitro development was also observed, as previously reported in *C. pepo* [15] and in other species such as melon and onion [8,51]. In addition, triploid plants were also detected. In *Lilium longiflorum* relatively high temperatures during meiosis and early in vitro culture are known to disturb meiotic divisions and increase the frequency of unreduced gamete [52]. Since comparable temperatures were applied during the induction phase in our system, they may likewise have perturbed embryo-sac development in *C. pepo* and favored endoreduplication or nuclear fusion, ultimately giving rise to triploid embryogenic cells. This phenomenon is generally attributed to premature endoreduplication in female gametophytic tissues [53], and has been shown to occur more frequently after cold pretreatment, thereby increasing the number of spontaneously DHs plants [54]. The relative proportion of haploids and DHs is known to vary with genotype and donor physiology [55]. The available evidence strongly supports the haploid origin of regenerants that initially showed haploid flow-cytometric profiles and later underwent spontaneous diploidization. All greenhouse plants displayed a uniform green zucchini phenotype, and no clear morphological traits distinguished materials with different ploidy histories; the only appreciable difference was vigor, as only the most vigorous plants set fruit and provided seed for 6 of the 8 lines, ensuring their maintenance. Even so, extending phenotypic assessment to larger regenerant sets, together with molecular marker or sequencing-based analyses, would offer a robust complementary approach to definitively confirm the gametophytic origin of plants that exhibited diploid profiles from the outset. In this study, no artificial chromosome-doubling treatments were required, as diploid plants arose spontaneously. These findings confirm that unpollinated ovary culture in *C. pepo* can yield both haploid and spontaneously doubled haploid plants of genuine gynogenetic origin.

## 4. Materials and Methods

### 4.1. Plant Materials and Growth Conditions

This study used two *Cucurbita* species: *Cucurbita pepo* (cv. Victoria F1, a dark green zucchini; HM Clause, Portes-lès-Valence, France) and *Cucurbita maxima* (cv. Zucchiolo F1, a yellow squash variety; Beyond Seeds, Almería, Spain). Both cultivars are commercial F1 hybrids. Plants were grown under controlled greenhouse conditions without pollinators at the El Bardo Experimental Station (36.81893, −2.23587, Almería, Spain) during the 2024–2025 autumn, winter, and spring growing cycles.

Female flowers were collected at two developmental stages: (a) one day before anthesis (1 DBA), and (b) at anthesis. Samples were collected early in the morning and immediately transferred to the laboratory in a portable cooler containing ice. Upon arrival, the flowers were stored at 4 °C for 24 h before ovary dissection.

### 4.2. Ovary Collection and In Vitro Culture Conditions

Petals and styles were carefully removed, and ovaries were subjected to an initial washing step with water and mild detergent to eliminate surface debris. After rinsing, the ovaries were peeled and transversely sliced into thin sections approximately 1–2 mm thick. Disinfection followed a two-step protocol: immersed in 70% ethanol for 1 min, followed by 30% (*v*/*v*) commercial bleach containing 3.7% (*w*/*w*) NaOCl (final available chlorine approximately 1.11%) for 15 min with constant agitation. Explants were then rinsed three times with sterile distilled water. Ten ovary slices were placed per 90 mm Petri dish, evenly spaced on the culture medium. All manipulations were performed under a laminar-flow hood.

### 4.3. Culture Medium and Hormonal Treatments

Nine induction media were used for the initial screening phase. The formulations were based on protocols reported in previous studies. All media consisted of Murashige and Skoog [56] basal salts and vitamins, supplemented with sucrose and plant growth regulators (Table 4). The pH of each medium was adjusted to 5.8 before adding Gelrite™ (5 g·L^−1^) and sterilization by autoclaving at 121 °C for 20 min. During the induction phase, cultures were incubated in darkness for 7 days at 35 °C and then transferred to 25 °C under a 16 h light/8 h dark photoperiod (approximately 2500 lx). All reagents used were obtained from Duchefa Biochemie (Haarlem, The Netherlands).

Four zeatin riboside (ZR)-based formulations were evaluated to optimize embryogenic induction and conversion (Table 5). These included adapted media from *S. melongena* and anther-culture protocols [33,58], and two modified versions developed in this study, differing in sucrose concentration and auxin balance.

### 4.4. Experimental Design and Data Collection

**Figure 7:**
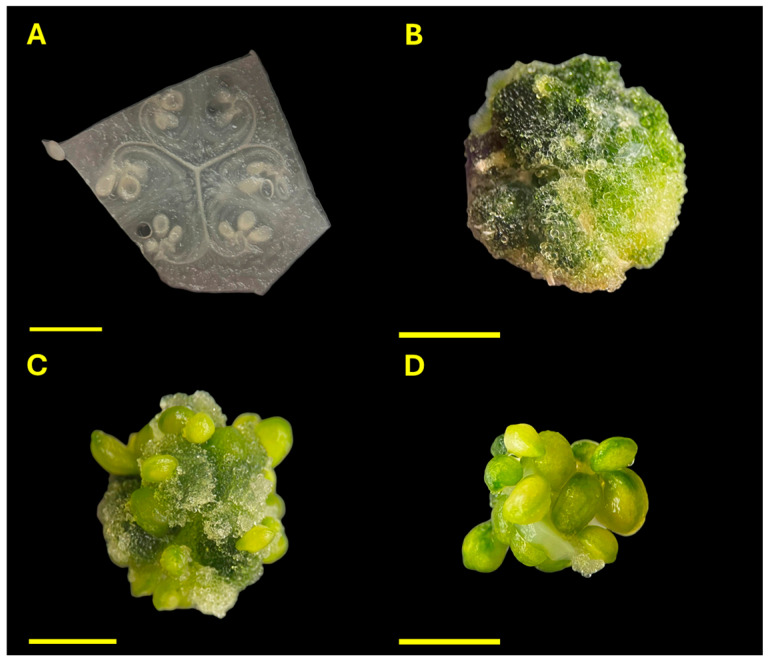
Morphological responses of ovary-derived explants of *Cucurbita pepo* after 30 days of culture. (**A**) No visible response; (**B**) Callus formation from somatic tissue; (**C**) Callus formation accompanied by the development of swollen ovules (SOs); (**D**) Direct formation of SOs without visible callus. Scale bars = 0.5 mm.

Morphological responses of ovary-derived explants were assessed after 30 days of culture using a qualitative scoring system with four predefined categories: (A) no visible response; (B) callus formation from somatic tissue; (C) Callus formation accompanied by the development of swollen ovules; and (D) formation of swollen ovules without visible callus (Figure 7).

The initial screening phase involved nine culture media; each tested with 100 explants. From day 45 onward, explants were monitored for the development of embryo-like structures (ELSs), which were quantified on a per-explant basis. Observations continued for a total period of 120 days.

In the ZR-based optimization phase, media were evaluated in three independent experimental sessions (60 explants per medium; 180 total). After excluding losses from contamination (minimum 138 valid explants), no significant differences were found between sessions, and the data were therefore pooled. Two quantitative variables were analyzed: (1) swollen ovular structures (SOs) per explant (day 30); and (2) embryos per explant.

### 4.5. Statistical Analysis

All statistical analyses were performed in Python 3.10 (Python Software Foundation, Wilmington, DE, USA) using SciPy, Pandas, and related libraries. Data distributions were tested for normality using the Shapiro–Wilk test. As most variables deviated from normality, non-parametric tests were applied. Morphological response categories (types A–D) were analyzed using chi-square (χ^2^) tests with Cramer’s V as effect size, followed by Dunn–Bonferroni post hoc comparisons. Quantitative variables (SOs and embryos per explant) were compared among media using the Kruskal–Wallis test with Dunn–Bonferroni pairwise adjustments. Associations between response type and embryogenic output were assessed using the Mann–Whitney U test. Data from the two sessions were statistically homogeneous and were therefore pooled. Statistical significance was set at *p* < 0.05.

### 4.6. Acclimatization and Greenhouse Transfer

Embryos at the cotyledonary stage, showing visible root primordia, were transferred to regeneration medium until autonomous plantlets were obtained. Acclimatization began when plantlets showed vigorous growth and well-formed roots. Each plantlet was transplanted into a small pot containing peat-based substrate, covered with a transparent plastic dome, and maintained at 25 °C under a 16 h light/8 h dark photoperiod. Humidity was gradually reduced over several days by partially lifting the domes to allow adaptation to ex vitro conditions.

When plants reached the 6–8 true-leaf stage and were fully acclimatized, ploidy analysis was performed on potted plants. Surviving acclimatized plants were transferred to the greenhouse and later grown under open-field conditions for self-pollination.

### 4.7. Flow Cytometry for Ploidy Verification

The ploidy level of regenerated plants was assessed by flow cytometry following acclimatization. Young leaf tissue (approximately 1 cm^2^) was collected from each plant for nuclear extraction and DAPI staining. Nuclear suspensions were prepared by mechanical homogenization in 500 µL of nuclear extraction buffer following the protocol of Doležel [59]. The suspension was filtered through a 30 µm nylon mesh and stained with 500 µL of DAPI staining solution.

Flow cytometric analysis was performed using a CyFlow^®^ Space flow cytometer (Sysmex, Hamburg, Germany) equipped with a fixed gain of 545. The G_0_/G_1_ peak of an internal diploid control was adjusted to 100 fluorescence units and used as the reference for determining the relative ploidy level of each sample. The presence of haploid, diploid, or higher-ploidy peaks was inferred from the position of sample peaks relative to the control. A second ploidy assessment was conducted one month later using a new leaf sample from each plant to confirm ploidy stability.

## 5. Conclusions

This study provides the first comprehensive demonstration that unpollinated ovary culture constitutes an efficient and reproducible route for gynogenetic haploid and doubled haploid induction in *Cucurbita pepo*. The use of ZR as the principal cytokinin proved decisive, promoting the formation of embryogenic swollen ovules and achieving the highest conversion efficiency among all media tested. This represents the first successful application of ZR in cucurbit gynogenesis and positions it as a physiologically balanced alternative to traditional cytokinins such as BAP or TDZ. The establishment of a standardized A–D morphogenic scale further enabled the early identification of competent explants, providing a reliable predictive framework for response assessment. Flow cytometric analyses confirmed the haploid origin of regenerants and revealed spontaneous diploidization events, validating the gametophytic nature of embryogenesis in *C. pepo*.

Beyond its immediate significance for zucchini, these findings open new avenues for testing ZR-based formulations across other *C. pepo* varieties, *Cucurbita* species and related genera, in combination with different auxin and carbohydrate regimes, to enhance embryogenic efficiency and genotype responsiveness. Future research should also focus on refining the hormonal and osmotic balance of the medium, exploring stress pre-treatments, and integrating molecular markers to track haploid origin and chromosomal stability. In this context, the homozygosity of the diploid regenerants will be confirmed in future work through the development and validation of specific molecular markers. Altogether, this work establishes a solid physiological and methodological foundation for haploid technology in *Cucurbita*, contributing to the acceleration of DH breeding pipelines in this economically relevant crop group.

## Figures and Tables

**Table 1:** Morphological responses of *C. pepo* ovary explants across eight culture media and two floral stages: 1 DBA and anthesis. Responses were classified as A (no response), B (callus), C (callus with swollen ovules), and D (swollen ovules). For each medium (n = 100 explants), the number of explants per response type, total embryos, and number of embryo-producing explants are shown. A control medium was tested but excluded due to complete non-responsiveness (100% type A).

Floral Development	Medium	Response				No. of Embryos	No. of Explants with Embryos
		A	B	C	D		
1 DBA	2007	6	28	37	29	27	17
	2018	20	43	28	9	4	3
	2020	10	21	21	48	5	4
	CU7	34	17	10	39	4	4
	CU8	4	14	18	64	2	2
	HYL6	13	59	26	2	0	0
	HYL12	27	7	18	48	3	2
	E6.2	7	12	22	59	50	29
At anthesis	2007	19	28	33	20	0	0
	2018	27	38	28	7	0	0
	2020	11	23	20	46	0	0
	CU7	40	16	9	35	0	0
	CU8	16	19	17	48	0	0
	HYL6	23	51	25	1	0	0
	HYL12	33	8	16	43	0	0
	E6.2	26	11	15	48	3	2

**Table 2:** Summary of swollen ovule (SO) structure induction in *C. pepo* ovary explants cultured in four zeatin riboside (ZR)-based media. Values show total number of explants, percentage of responsive explants, mean ± SD number of SOs per explant, and number of SOs per 100 explants.

Medium	No. of Explants	% Responsive Explants	SOs per Explant (Mean ± SD)	SOs/100 Explants
E6	138	62.3%	4.00 ± 4.18	400.00
E6.1	165	84.9%	5.64 ± 3.68	564.24
E6.2	180	85.0%	6.09 ± 3.54	608.89
E6.3	155	81.9%	6.01 ± 4.16	600.65

**Table 3:** Embryo production from ovule-like structures induced in *C. pepo* ovary explants cultured in ZR-based media. Values show total embryos, mean ± SD and median embryos per explant, embryos per 100 explants, percentage of explants with ≥1 embryo, and conversion rate.

Medium	Total Embryos	Embryos/Explant (Mean ± SD)	Embryos/100 Explants	% Explants ≥ 1 Embryo	Conversion Rate (%)
E6	103	0.75 ± 1.42	74.64	32.6%	18.7%
E6.1	160	0.97 ± 1.54	96.97	44.9%	17.2%
E6.2	29	0.16 ± 0.37	16.11	16.1%	2.7%
E6.3	60	0.39 ± 0.73	38.71	27.1%	6.4%

**Table 4:** Composition of culture media used for initial screening, regeneration and rooting. All media were prepared with Murashige and Skoog (MS) basal salts and vitamins [56]. All concentrations are expressed in mg·L^−1^, except sucrose (g·L^−1^). “-” indicates the absence of the component.

Medium	Sucrose	BAP	IAA	KT	NAA	TDZ	ZR	2,4-D	PCM
Induction									
2007 [16]	30	-	-	1	-	-	-	1	-
2018 [36]	30	4	-	-	0.05	0.1	-	-	-
2020 [23]	30	1	-	-	-	-	-	-	-
CU7 * [57]	120	-	-	5	-	-	-	5	-
CU8	120	-	-	5	-	-	-	-	2
HYL6 [30]	60	-	-	-	-	0.5	-	0.2	-
HYL12 * [30]	120	-	-	-	-	0.5	-	0.2	-
E6.2 * [33]	120	-	-	-	3	-	1	-	-
A	30	-	-	-	-	-	-	-	-
Others									
Regeneration	30	1	0.01	-	-	-	-	-	-

* Modified formulations are denoted by an asterisk.

**Table 5:** Composition of ZR-based culture media used for the optimization of embryogenic response in *Cucurbita pepo*. All concentrations are expressed in mg·L^−1^, except sucrose (g·L^−1^). “-” indicates the absence of the component.

Medium	MS	Sucrose	ZR	NAA
E6 [58]	2.2	15	2	-
E6.1 [33]	4.4	30	1	3
E6.3	4.4	30	2	-
E6.2	4.4	120	1	3

## Data Availability

All data are presented within the article.

## Supplementary Materials

The following supporting information can be downloaded at: https://www.mdpi.com/article/10.3390/plants14243733/s1, Figure S1: Morphogenic response of *Cucurbita maxima* ovary explants after 30 days of culture; Figure S2: Heatmap of similarity among culture media tested in the initial screening of *Cucurbita pepo* ovary cultures; Figure S3: Standardized residuals from the chi-square test of morphogenic responses in *Cucurbita pepo* ovary cultures. Figure S4: Comparison of field-grown plants showing contrasting vigor. The plant on the left exhibits high vigor, whereas the plant on the right shows reduced vigor.

## Abbreviations

The following abbreviations are used in this manuscript:

1 DBA	One day before anthesis
2,4-D	2,4-Dichlorophenoxyacetic acid
BAP	6-Benzylaminopurine
DH	Doubled haploid
ELS	Embryos-Like Structure
IAA	Indole-3-acetic acid
KT	Kinetin
NAA	Naphthaleneacetic acid
SOs	Swollen ovules
PCM	Picloram
TDZ	Thidiazuron
Z	Zeatin
ZR	Zeatin riboside

## Appendix A

**Table A1:** Distribution of *Cucurbita pepo* ovary explants according to morphological response type (A–D) and culture medium. Each cell shows the number of explants and the total number of embryos obtained (explants|embryos). Totals correspond to the sum per medium and across all responses.

Medium	Response|Embryos				Total Explants	Total Embryos
	A	B	C	D		
E6	12|0	20|0	33|30	27|39	92	69
E6.1	0|0	16|0	48|25	46|77	110	102
E6.2	8|0	7|0	23|1	82|14	120	15
E6.3	3|0	17|0	42|15	41|22	103	37
Total	23|0	60|0	146|71	196|152	425	223
