# Supplementary figures and images for: Doubled Haploid Production in Cucurbita pepo L. Through Ovary Culture
Source: Plants (Basel). 2025 Dec 8;14(24):3733. doi: 10.3390/plants14243733 (PMC12737043; doi:10.3390/plants14243733)

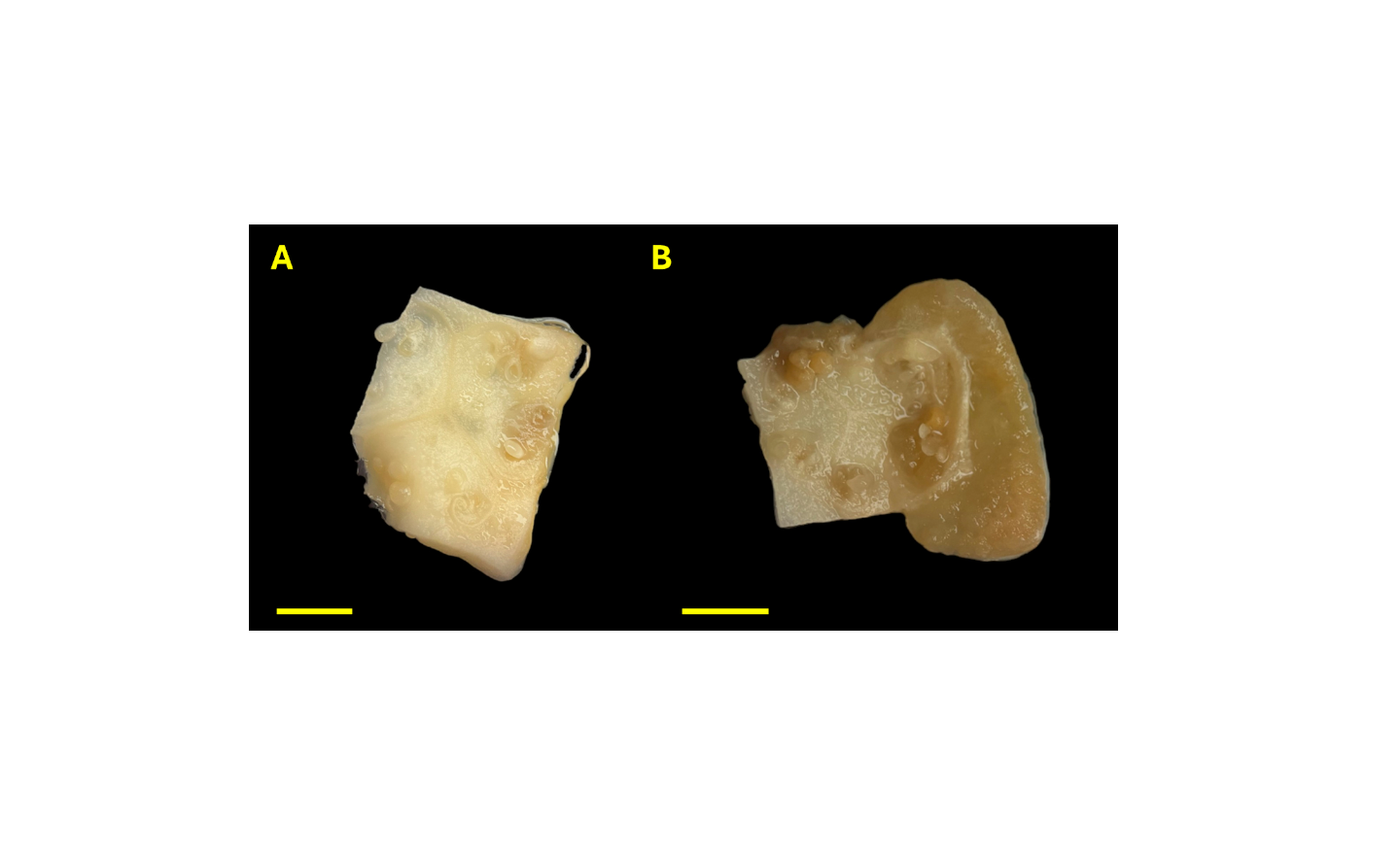

Supplement: Supplementary file 1 [file plants-14-03733-s001.zip › Figure_S1.png]

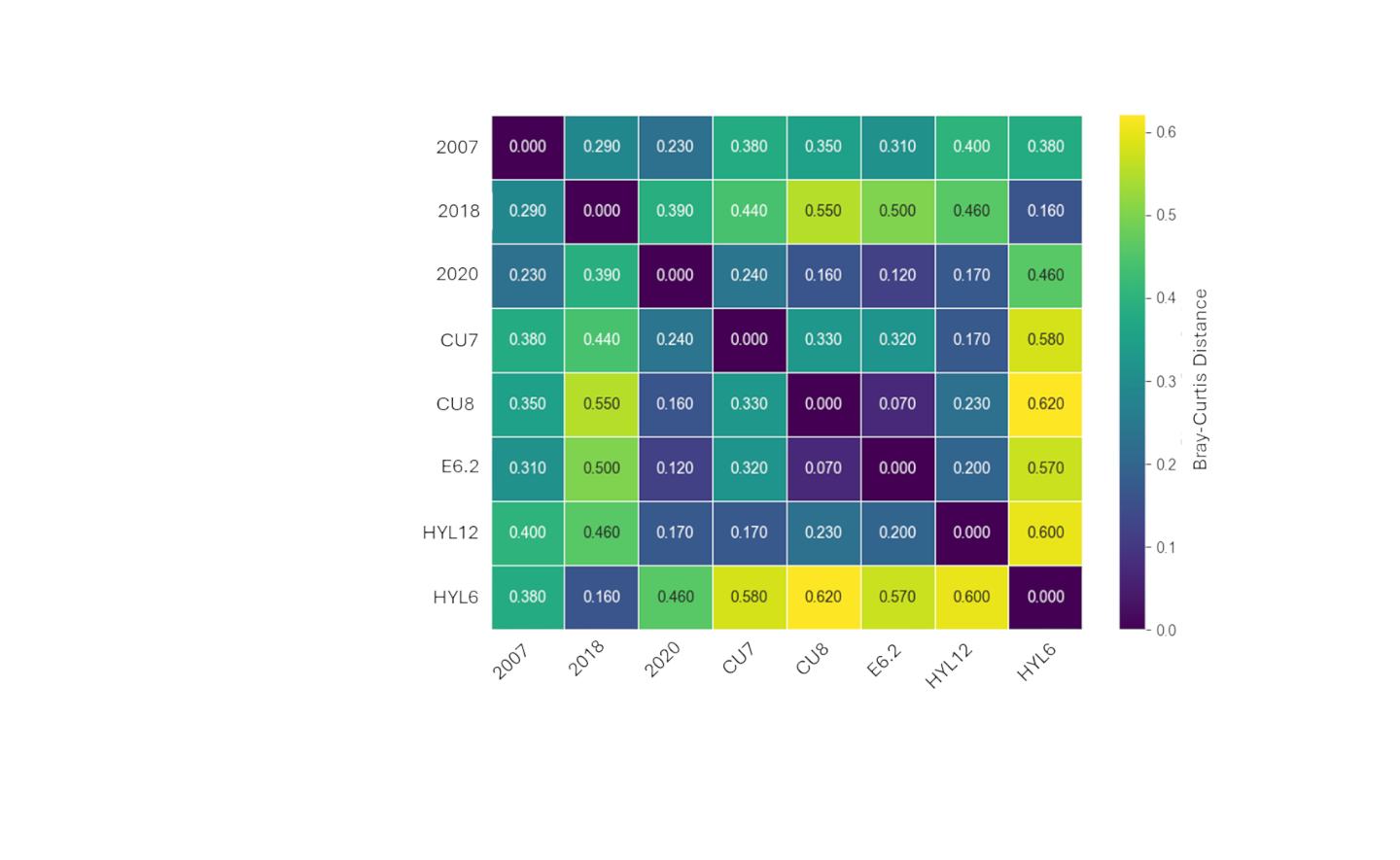

Supplement: Supplementary file 1 [file plants-14-03733-s001.zip › Figure_S2.png]

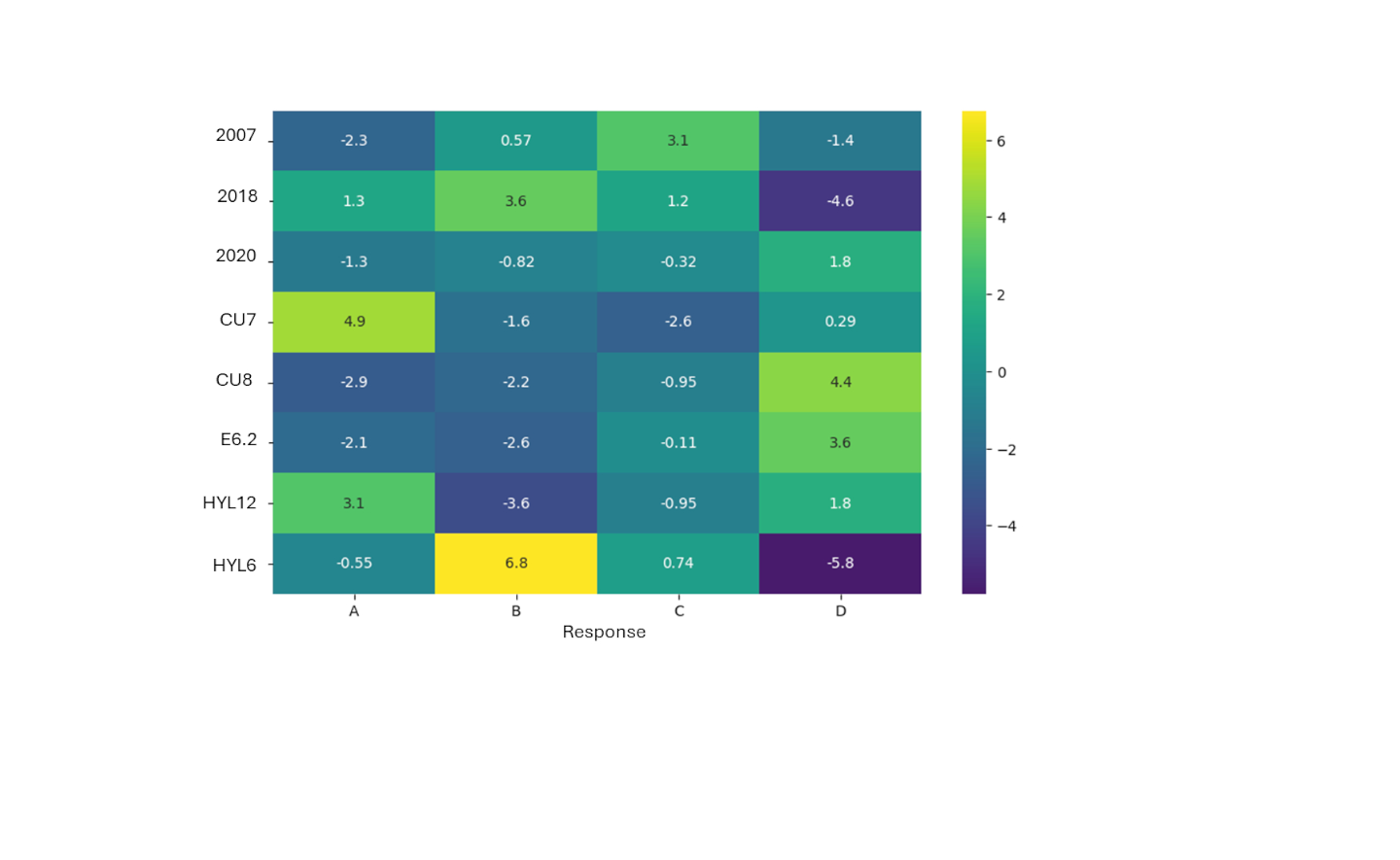

Supplement: Supplementary file 1 [file plants-14-03733-s001.zip › Figure_S3.png]

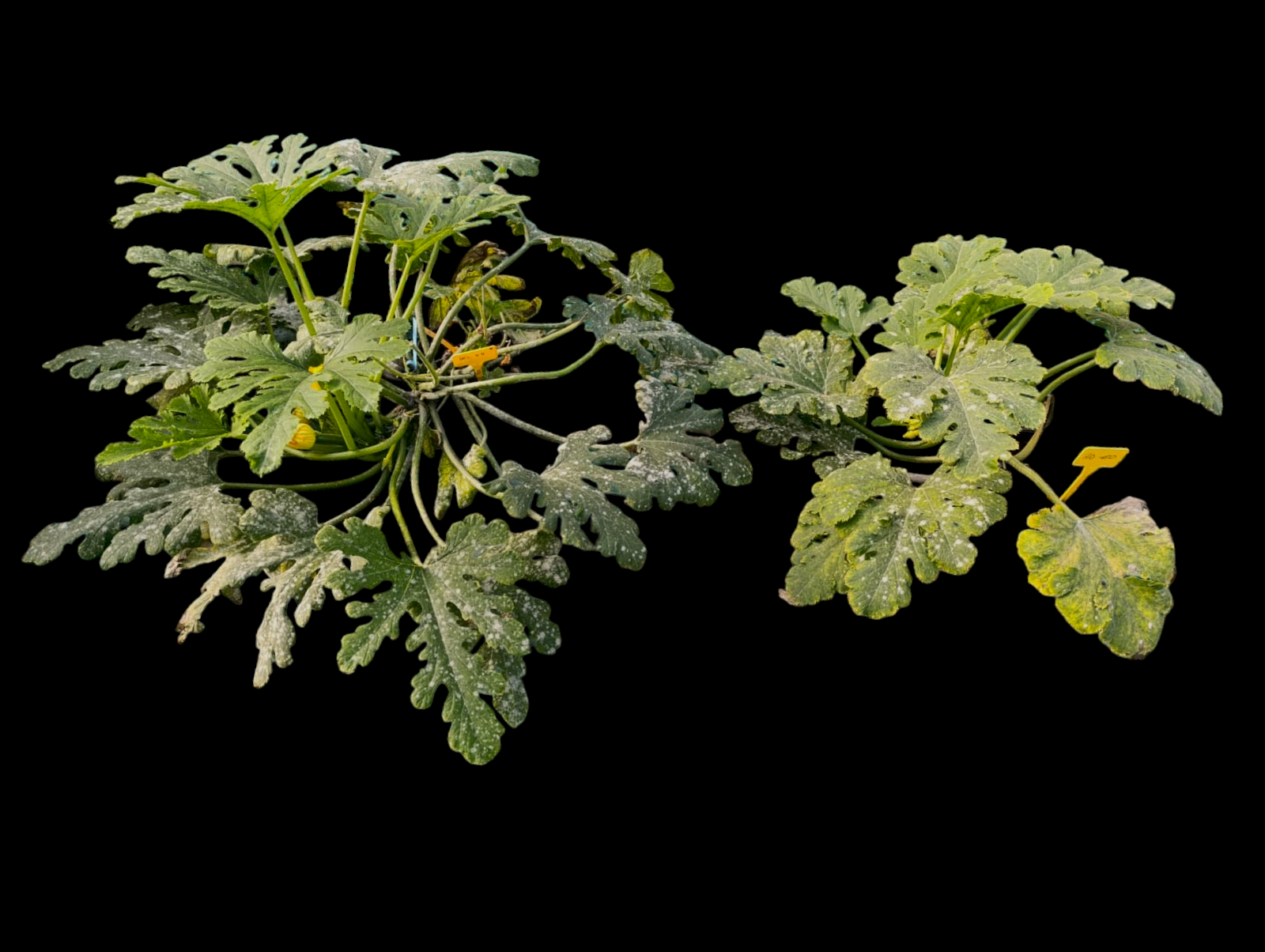

Supplement: Supplementary file 1 [file plants-14-03733-s001.zip › Figure_S4.1.png]

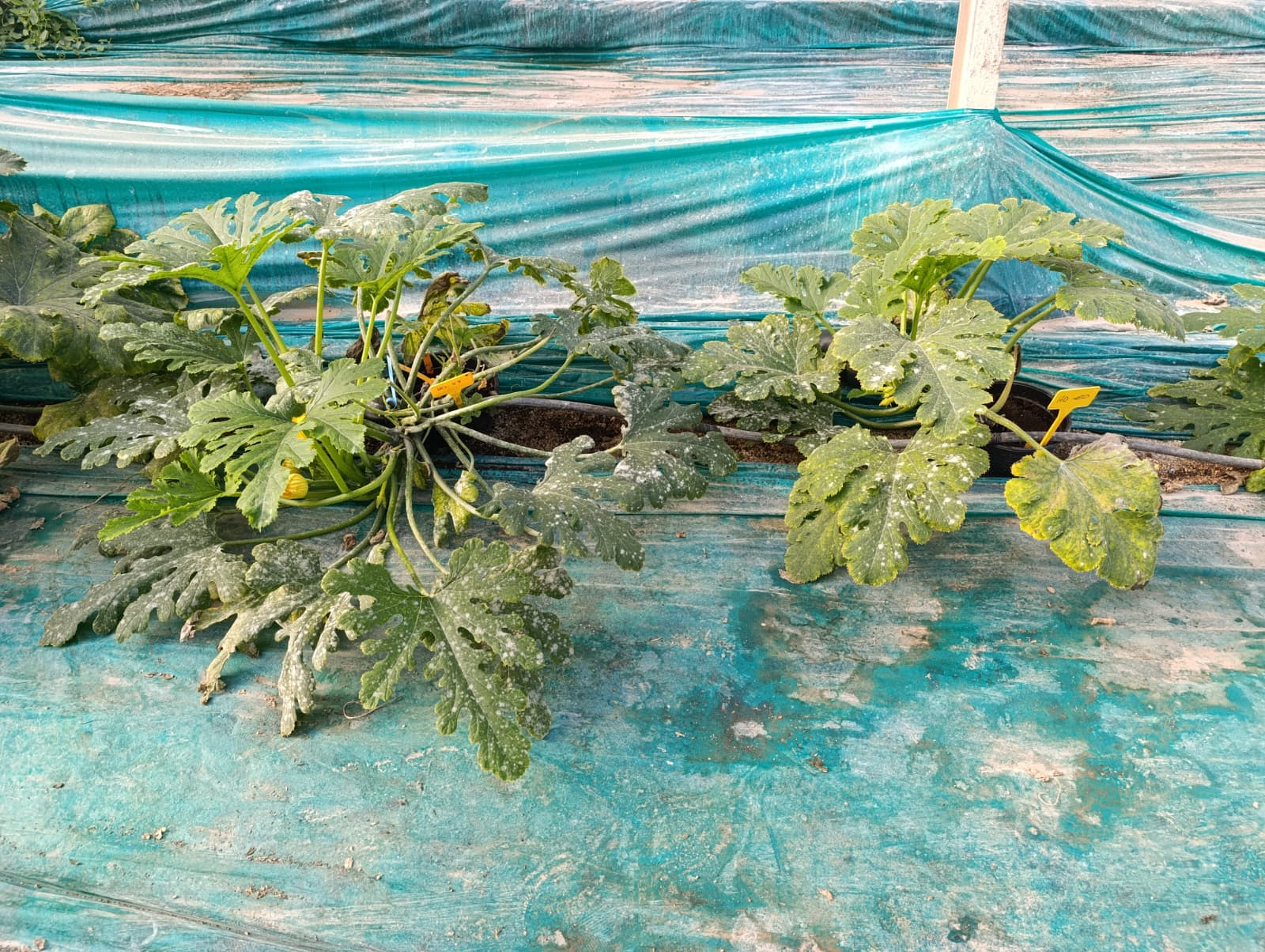

Supplement: Supplementary file 1 [file plants-14-03733-s001.zip › Figure_S4.2.png]
